# Criterion Validity and Psychometric Properties of a Malay Version of the Short Multidimensional Inventory Lifestyle Evaluation-Confinement (SMILE-C) in a Sample of University Staff with Weight Problems

**DOI:** 10.3390/ijerph181910410

**Published:** 2021-10-03

**Authors:** Nor Ba’yah Abdul Kadir, Wan Nur Khairunnisa Ismail, Nurul-Azza Abdullah, Rusyda Helma, Siti Jamiaah Abdul Jalil, Arena Che Kasim, Suzana Mohd Hoesni, Mohd Rizal Abdul Manaf

**Affiliations:** 1 Centre for Research in Psychology and Human Well-Being, Faculty of Social Sciences and Humanities, Universiti Kebangsaan Malaysia, Bangi 43600, Malaysia; nisaismail281@gmail.com (W.N.K.I.); nurulazza@ukm.edu.my (N.-A.A.); rusyda_h@ukm.edu.my (R.H.); arena@ukm.edu.my (A.C.K.); smh@ukm.edu.my (S.M.H.); 2 Department of Dakwah and Leadership, Faculty of Islamic Studies, Universiti Kebangsaan Malaysia, Bangi 43600, Malaysia; sitijamiaah82@ukm.edu.my; 3 Department of Community Health, Faculty of Medicine, Universiti Kebangsaan Malaysia, Kuala Lumpur 56000, Malaysia; mrizal@ppukm.ukm.edu.my

**Keywords:** assessment scales, instrumental study, lifestyle changes

## Abstract

The aim of this study was to validate the Short Multidimensional Inventory Lifestyle Evaluation-Confinement (SMILE-C) in a Malaysian context. The SMILE-C, which is a respondent-generated instrument, was used to ask participants questions on their lifestyle during the COVID-19 pandemic. The indices of seven sub-scores were then calculated. A total of 121 university staff members completed the Malay version of the SMILE-C as well as instruments for measuring well-being, family life satisfaction, mindfulness and awareness, work engagement, and quality of life. The Cronbach’s alpha values and Pearson correlation coefficients were satisfactory in this initial validation of the instrument. The SMILE-C showed positive correlations with all the variables being studied. The results supported the criterion-related validity and psychometric properties of the Malay version of the SMILE-C as an instrument for assessing lifestyle changes during the COVID-19 pandemic.

## 1. Introduction

The COVID-19 pandemic is causing life-altering problems for people worldwide [1,2,3]. There is a growing use of the terms “social distancing” [4] and “self-isolation” [5]. As people fight to adapt, confinement has had a huge influence on the lives of people and has rapidly and severely disrupted their daily routine and lifestyle [6,7,8,9]. Long periods of social isolation can lead to boredom and anxiety, thereby, resulting in a strong tendency to overeat, particularly high-calorie “comfort foods” [9,10]. In addition, prolonged confinement at home can lead to more time spent monitoring and less time spent outside, thus, giving rise to a more sedentary behavior [11,12,13,14]. Furthermore, if people are confined to their homes and their daily routines are interrupted, these can also affect their sleep patterns and quality of life [15]. To date, the primary instrument used for measuring changes, such as in eating, physical activities, and sleep patterns, in relation to the COVID-19 pandemic is the Short Multidimensional Inventory Lifestyle Evaluation-Confinement (SMILE-C) [16]. The original SMILE is a 43-item self-rated questionnaire consisting of seven domains (diet and nutrition, substance abuse, physical activity, stress management, restorative sleep, social support, and environmental exposures). An initial draft consisted of 48 items divided into six lifestyle domains (diet, physical activity, sleep, interpersonal relationships, work performance, safety, and access to health care). A few refinements were made; thus, some domains remained whilst another two domains were removed. The second draft consisted of 94 items, after redefined domain on health, only 69 items remained. Following careful revision, the research group removed 12 items and maintained the seven lifestyle domains. The experts review committee then suggested remaining at 43 items. The original SMILE was used to collect data and refined during the pandemic self-isolation/confinement. Using principal component analysis and factorial analysis, only 27 items remained. This short version of SMILE is called SMILE-C [16]. The SMILE-C was developed for a multidimensional and comprehensive assessment of a healthy lifestyle over the past 30 days. Response options are measured according to a 4-point Likert scale, and the final score is obtained as the sum of all the questions (some questions are reverse scores). The higher the score, the healthier the lifestyle (scores range from 27 to 108). The SMILE-C shows an overall Cronbach’s alpha value of 0.75 and Kaiser–Meyer–Olkin measure of 0.77 [16]. This scale was simultaneously developed in three languages: English, Spanish, and Portuguese [16,17]. 

As it is a new self-report measure, the criterion-related validity and psychometric properties of the SMILE-C has yet to be tested among Malaysians with weight problems. Given its very broad use for the general population and those with weight problems, it is important that the psychometric properties of the SMILE-C are well understood. Therefore, the objective of this study was to evaluate the criterion-related validity and the psychometric properties of the SMILE-C in a sample of university staff with weight problems.

## 2. Materials and Methods

### 2.1. Participants

A quasi-experimental design was used, where the participants were subjected to the intervention for 12 months. The research was designed and conducted in accordance with the Consolidated Standards of Reporting Trials 2010 statement, with extensions to randomized pilot and feasibility trials, where applicable [18].

The inclusion criteria included an age range of between 20 and 59 years, BMI of 25 and above, no chronic disease, and no history of bariatric surgeries. The exclusion criteria included pregnancy, history of using weight-loss pills, and consumption of supplements. This study involved two stages data collection. In stage one, participants were required to complete a self-administered questionnaire that consisted of items on socio-demographic characteristics (e.g., gender, age) and health status. The description for this study can be found elsewhere [19]. In stage two, participants were asked to complete questions on quality of life, mindfulness, work engagement, family life satisfaction, subjective well-being, and lifestyle. Mindfulness, work engagement, subjective well-being, and quality of life variables were measured before the implementation of Movement Control Order (MCO) due to pandemic COVID-19 and 12 months after the first contact. Meanwhile SMILE-C and adaptation (was not analyzed in this paper) were only measured at the end stage of this study.

Of the 538 university staff members in home confinement, a total 300 were not eligible to be selected due to normal BMI (*n* = 217) and chronic illness (*n* = 83). Of 235 eligible participants, only 121 of them returned the completed questionnaires. We used various ways to increase the response rate. These included soft reminders via emails, WhatsApp group, and personal messages. The participants comprised 34 males, 82 females, and 5 participants who did not state their gender, majority were of Malay ethnicity (99%), and Muslims (99%). The mean age of the participants was 41.6 years and the SD was 6.51. In terms of occupational status, only 8 participants were academic staff, and the rest were non-academic staff (management and professional, *n* = 18; support and administration, *n* = 95).

The minimum sample size requirement was determined based on 5:1 ratio, the desired level was between 15 and 20 observations for each independent variable [20,21]. Thus, the sample size of this study can be considered as acceptable.

### 2.2. Measures

The World Health Organization Quality of Life-BREF (WHOQOL-BREF), the Well-being Scale (WeBS), the Satisfaction with Family Life Scale (SWFL), the Mindfulness Attention Awareness Scale (MAAS), and the Utrecht Work Engagement Scale UWES were used as independent criteria that reflected SMILE-C scores. This procedure examined the relationships between SMILE-C and well-established measures to see if there is a relationship between the scores. No ‘‘gold standard’’ was used to check sensitivity and specificity of the SMILE-C but evaluation was made to other measures that appear reasonable.

The WeBS was designed to measure the overall well-being in terms of the physical, financial, social, hedonic, and eudaimonic well-being [22]. The scale consists of 29 items on a Likert-type scale (strongly disagree to strongly agree). Overall, the WeBS scores and five domain-specific subscale scores demonstrated adequate to excellent internal consistency reliability and construct validity. The mean differences in the overall well-being and its five subdomains are presented for different ethnic groups. The WeBS is a reliable and valid measure of multiple aspects of well-being.

The SWFL modelled after the Satisfaction with Life Scale, was designed to assess an individual’s global judgment of family satisfaction, which is theoretically predicted to depend on a comparison of family life circumstances with one’s own standards and expectations [23]. The scale consists of five items on a Likert-type scale (strongly disagree to strongly agree). Previous studies have shown that across all samples a consistent unidimensional factor structure is maintained, with a Cronbach’s alpha ranging from 0.94 to 0.79 [22]. Evidence of its usability, criterion, and construct validity has also been established. The SWFL scale provides a brief, psychometrically sound, and widely applicable option for measuring satisfaction with family life.

The MAAS was developed to measure people’s tendency to be mindful of moment-to-moment experiences [24]. The MAAS consists of 15 items on a Likert-type scale (strongly disagree to strongly agree). Thus, the instrument focuses on the presence or absence of attention and awareness of what occurs in the present. This scale has been shown to relate to various aspects of well-being and how effectively people deal with stressful life events [24]. It has been found to have good internal consistency, with alpha values of 0.82 and 0.87 in student and adult samples, respectively. The MAAS demonstrates convergent and discriminant correlations in the expected direction with other measures, such as the NEO-PI, NEO-FFI, the Mindfulness/Mindlessness Scale (MMS), Beck’s Depression Inventory (BDI), Rosenberg’s Self-Esteem Scale, and the State-Trait Anxiety Inventory (STAI).

The UWES is used to measure vigor, dedication, and absorption [25]. The Malay version of the UWES consists of nine items, and all the items are scored on a seven-point frequency rating scale ranging from 0 (never) to 6 (always). A total score (which can range from 0 to 54) is calculated from all nine items, and the average score, which is obtained by dividing the total sum of the items by the number of items, provides the mean of the overall work engagement. Although the original UWES-9 consists of three subscales, comprised of the main components of vigor, dedication, and absorption, this study examined work engagement as a single-factor structure. This was in line with the guidelines for tool usage, as verified in the Malaysian context and supported by previous research that examined the factor structure of the UWES.

The WHOQOL-BREF is a 26-item self-report measure consisting of four domains, namely, physical health (7 items), psychological health (6 items), social relationships (3 items), and environmental health (8 items). It also contains QOL and general health items. Each individual item of the WHOQOL-BREF is scored from 1 to 5 on a response scale, which is stipulated as a five-point Likert-type scale. The physical health domain includes items on mobility, daily activities, functional capacity, energy, pain, and sleep. The psychological domain measures include self-image, negative thoughts, positive attitudes, self-esteem, mentality, learning ability, memory concentration, religion, and mental status. The social relationships domain contains questions on personal relationships, social support, and sex life. The environmental health domain covers issues related to financial resources, safety, health, and social services, living physical environment, opportunities to acquire new skills and knowledge, recreation, general environment (noise, air pollution, etc.), and transportation. The report showed that the WHOQOL-BREF has good psychometric properties [26].

### 2.3. Procedures

We used Braken and Barona’s procedure for translating and validating in cross-cultural assessments [27]. The English version of the SMILE-C scale was translated into Malay by two qualified bilingual translators and who are sufficiently educated to have familiarity with the concepts in the field. A blind translation of a translated version back into the English language was performed. The translators and the principal investigator reconciled any discrepancies between the translated versions and the English SMILE-C. The individuals who conducted the back-translation have no prior knowledge of the scale being translated. The translated versions were then checked by two subject matter experts (SMEs or a bilingual review committee), who are bilingual English and Malay speakers and proficient in the field. The SMEs were blinded to the English version of SMILE-C. Based on the feedback of the SMEs, minor changes were made to enhance the lucidity of the translated version, which was then compared again to the English version.

The pilot study was conducted to check internal consistency of the items. A total of 43 participants completed the questionnaires [28]. Result showed that all items were acceptable apart item 6 (corrected-item total correlation 0.00). As suggested by the review committee, this item was removed from the study due to religion sensitivity. On top of that, most of the participants in this study are Muslims. Drinking alcohol is forbidden in Islam. Most participants responded “never” to item 6: “Do you drink 5 or more doses (men) or 4 or more doses (women) of alcoholic beverages on a single occasion, which means within 2 h? (1 dose of alcohol = 1 glass of beer OR 1 glass of wine OR 1 shot of spirit (such as rum, vodka, whisky, tequila or gin))”. The value of Cronbach’s alpha for 26 items was 0.74.

The university staff responded to the self-administered instruments via a Google form between March and April 2021. Participants were briefed on the purpose of the study and written consent was obtained. All the participants who responded to the questionnaire were informed that all the data gathered would remain anonymous and confidential. Ethical approval was obtained from the Research Ethics Committee, Universiti Kebangsaan Malaysia.

### 2.4. Statistical Analysis

For all the tests, a significance level of 0.05 was considered, and the statistical software, SPSS version 26, was used. The internal consistency reliability was estimated using the Cronbach’s alpha coefficient and item-total correlation. Criterion validity is the “degree to which there is a relationship between a given test score and performance on another measure of particular relevance, typically referred to as a criterion” [29,30] or to the degree to which a test score is related to a meaningful outcome or criterion of interest [31]. The criterion-related validity was tested by seeking relationships between the SMILE-C and quality of life, well-being, family life satisfaction, and mindfulness and awareness. These relationships were mentioned in several previous studies [32,33,34,35,36,37,38].

Two methods are used to examine construct validity. First, convergent validity is the extent to which a construct measured in different ways yields similar results. Specifically, it is the “degree to which scores on a studied instrument are related to measures of other constructs that can be expected on theoretical grounds to be close to the one tapped into by this instrument” [39,40]. Inter-correlations were used to examine the convergent validity between the dimensions of the SMILE-C and the dimensions of the WHOQOL-BREF. The convergent validity of a construct is provided by the extent to which a new instrument correlates highly with other variables in measuring the same construct [41]. It can be invalidated by correlations that are too low or weak with other tests which are intended to measure the same construct.

Second, discriminant validity is the extent to which a measure is novel and not simply a reflection of some other construct [42]. Specifically, it is the “degree to which scores on a studied instrument are differentiated from behavioural manifestations of other constructs which, on theoretical grounds, can be expected not to be related to the construct underlying the instrument under investigation” [43]. The discriminant validity was tested by seeking relationships between the SMILE-C and work engagement.

## 3. Results

The analysis of internal consistency yielded a Cronbach’s alpha of 0.92 for the Well-being Scale, 0.85 for the Satisfaction with Family Life Scale, 0.95 for the Mindfulness and Awareness Scale, 0.96 for the Utrecht Engagement Scale (UES), 0.91 for the WHOQOL-BREF, and 0.83 for the Short Multidimensional Inventory Lifestyle Evaluation-Confinement (Table 1).

The item-total correlation between the full score of the SMILE-C and 26 items of the SMILE-C were used to examine the construct validity. A modest correlation was found in this analysis, and only two items were not significant (Table 2).

A criterion-related validity was then performed to see if the SMILE-C correlated highly with other variables that it ought to correlate with [40,41]. Hence, the SMILE-C was expected to show a high correlation with quality of life, well-being, satisfaction with family life, and mindfulness and awareness. The results showed that the correlation coefficients were positive and significant for all the variables being studied (Table 3).

A further analysis was carried out to examine the convergent validity by dimension of the SMILE-C and WHOQOL-BREF. The results showed that the correlation coefficients were positive (negative) and significant for the dimensions of SMILE-C and WHOQOL-BREF (Table 4). The values of *r* indicate a weak (0 and 0.3; 0 and −0.3), moderate (0.3 and 0.7; 0.3 and −0.7), and strong (0.7 and 1.0; −0.7 and −1.0) linear relationship among variables. However, a weak correlation may indicate that these factors are not a good measure of lifestyle changes during the COVID-19 pandemic among university staff with weight problems.

The discriminant validity was estimated by comparing the scores on the SMILE-C with the UES. It was expected that there would be no or low correlations between the SMILE-C and UES during the lockdown due to the COVID-19 pandemic. Table 5 shows that the correlation coefficients were positive and significant (not significant) between the dimensions of SMILE-C as well as negative and significant (not significant) between substance use in the SMILE-C and UES. The values of *r* indicated a weak positive (negative) linear relationship between the SMILE-C and UES.

## 4. Discussion

The objective of this study was to evaluate the criterion-related validity and psychometric properties of the SMILE-C by investigating the link between well-being, family life satisfaction, work engagement, and quality of life in a sample of university staff. Overall, the analyses provided initial evidence of the criterion-related validity of the SMILE-C in a sample of university staff. The results indicated that lifestyle changes during the COVID-19 pandemic, particularly throughout the home isolation, were significantly associated with well-being, family life satisfaction, and quality of life. The SMILE-C dimensions were also significantly correlated to work engagement. Therefore, it is best to use a couple of justifications to describe the significant positive correlation between SMILE-C and work engagement. As stated in the findings, the SMILE-C discussed the importance of the degree of crafting and modifying lifestyle. First, it is important to note here that the data collection was conducted during a national lockdown, whereby employees were allowed to work from home. Such an allowance gave the employees extra room to manage their time well to balance work and household chores. The lockdown also provided employees with more time as they were able to cut down on their commuting hours and unnecessary stress during the journey. Secondly, as the employees did not have to comply with strict requirements, such as being at work during office hours, there was also a reduction in their stress and an increase in their autonomy [42]. The most important foundation of work engagement is the autonomy that is nurtured by the organization [43]. This autonomy will then increase the employees’ self-motivation and self-efficacy, thereby leading to work engagement [44]. Employees who are confident and feel that they can contribute to the development of the workplace feel the enjoyment and excitement of performing well at work.

Overall, the data suggested that the SMILE-C may be a useful measure for assessing well-being, family life satisfaction, mindfulness and awareness, work engagement, and quality of life. Although the data indicated a general association with quality of life, three dimensions of the SMILE-C (substance use, physical activities, and environmental exposure) did not correlate with the dimensions of quality of life. These findings can best explain these dimensions and may indeed be somewhat independent.

The limitations of this study should be acknowledged. First, it should be noted that the findings were based on a selected group of university staff with weight problems. Thus, it is important for future studies to examine lifestyle changes due to the COVID-19 pandemic in the general population and in groups with specific patients with Type 2 diabetes. Second, this study was limited to a small sample size to run an advanced inferential statistical analysis, such as structural equation modelling. Third, the SMILE-C was measured only once, and therefore, no statement could be made about the possible role of the causal relationships of the SMILE-C. These limitations notwithstanding, the results of this study provided initial support for the criterion-related validity and psychometric properties of the SMILE-C when administered to university staff with weight problems. Future research is suggested to conduct test-retest reliability to ensure that SMILE-C is representative and stable over time [39,45].

Clearly, the findings of this study have several practical and theoretical implications. For instance, at the practical level, the use of the SMILE-C self-report measures may be particularly useful to consider during a crisis. At the theoretical level, it fulfils the need for a comprehensive model for improving the quality of life that incorporates individual differences in lifestyle changes during the COVID-19 pandemic, particularly during home isolation.

## 5. Conclusions

The positive results of the criterion-related validity and psychometric properties of the Malay version of the SMILE-C provide a foundation for a larger-scale investigation of this instrument as a culturally appropriate tool to assess lifestyle changes during the COVID-19 pandemic among Malaysians with weight problems and to further explore its properties.

## Figures and Tables

**Table 1:** Internal consistency reliability for measures used in this study.

Measures	Cronbach’s Alpha
Well-being Scale (WeBS)	0.92
Satisfaction with Family Life Scale (SWFL)	0.85
Mindfulness Attention Awareness Scale (MAAS)	0.95
Utrecht Engagement Scale (UES)	0.96
World Health Organization Quality of Life-BREF (WHOQOL-BREF)	0.91
Short Multidimensional Inventory Lifestyle Evaluation-Confinement (SMILE-C)	0.83

**Table 2:** Item-total correlation between items of SMILE-C and full score of the SMILE-C.

Items for SMILE-C	*r* (Total SMILE-C)
1. Do you eat processed food (frozen food, such as pizza, French fries, puff pastries, deep-fried food, and canned foods)?	0.00
2. Do you eat fast food, high-calorie sweet or fatty foods when you are stressed or sad?	0.29 **
3. Do you eat healthy foods, such as fresh fruits, fresh vegetables, wholegrains, legumes, or nuts?	0.47 **
4. Do you keep a regular meal schedule?	0.60 **
5. Do you share your main meals with friends or family?	0.55 **
6. Do you smoke tobacco (cigarette, electronic cigarette, cigar, pipe, smokeless tobacco)?	0.19 *
7. Do you use marijuana or hashish?	0.32 **
8. Do you use other drugs (cocaine, crack, amphetamines, ecstasy, opioids without medical prescription, and others?	0.26 **
9. Do you exercise for at least 30 min daily (or 150 min) a week?	0.24 **
10. Do you make time to relax?	0.47 **
11. Do you use any strategy or psychological support to deal with stress (for instance meditation, mindfulness, or psychotherapy)?	0.27 **
12. Do you use physical strategies to deal with stress (for instance yoga, tai chi, exercise)?	0.31 **
13. Do you practice a faith or religion?	0.49 **
14. Do you feel that your life has meaning?	0.58 **
15. Do you feel grateful for the life you have?	0.57 **
16. Do you manage to sleep between 7 and 9 h per night?	0.59 **
17. Do you feel rested with the number of hours you sleep?	0.61 **
18. Do you maintain a regular sleep schedule?	0.57 **
19. Do you use sleeping pills?	0.33 **
20. Do you interact with friends and/or relatives?	0.62 **
21. Do you feel that you are part of a group of friends, the community or society?	0.68 **
22. Do you have someone you trust who listens to your problems or concerns?	0.66 **
23. Do you have someone to help with everyday chores (for instance cooking, housekeeping, shopping)?	0.43 **
24. Do you enjoy your leisure time?	0.70 **
25. Do you make yourself available to support your significant ones?	0.68 **
26. Do you spend time on a computer/smartphone within one hour of going to sleep?	0.02

** Correlation is significant at the 0.01 level (2-tailed); * correlation is significant at the 0.05 level (2-tailed).

**Table 3:** Criterion-related validity between SMILE-C and other measures.

Measures	SMILE-C
Well-being Scale (WeBS)	0.60 **
Satisfaction with Family Life Scale (SWFL)	0.50 **
Mindfulness Attention Awareness Scale (MAAS)	0.45 **
World Health Organization Quality of Life-BREF (WHOQOL-BREF)	0.62 **

** Correlation is significant at the 0.01 level (2-tailed).

**Table 4:** Examining convergent validity by dimension of SMILE-C and WHOQOL-BREF.

Dimensions of SMILE-C	1	2	3	4	5	6	7	8	9	10	11
1. Diet and nutrition	-										
2. Substance use	0.08	-									
3. Physical activities	0.25 **	−0.17	-								
4. Stress management	0.32 **	0.13	0.12	-							
5. Restorative sleep	0.46 **	0.08	0.10	0.41 **	-						
6. Social support	0.50 **	0.15	0.06	0.58 **		-					
7. Environmental exposures (screen time/outdoor time)	0.18	−0.05	0.06	−0.11	−0.06	−0.17	-				
8. Physical QOL	0.48 **	−0.05	0.15	0.34 **	0.49 **	0.54 **	−0.01	-			
9. Psychological QOL	0.31 **	−0.19 *	0.10	0.36 **	0.33 **	0.45 **	0.03	0.62 **	−		
10. Social relationship QOL	0.41 **	−0.12	0.24 **	0.40 **	0.55 **	0.52 **	−0.02	0.73 **	0.64 **	−	
11. Environmental QOL	0.39 **	−0.11	0.15	0.44 **	0.36 **	0.59 **	−0.06	0.66 **	0.70 **	0.73 **	−

** Correlation is significant at the 0.01 level (2-tailed); * correlation is significant at the 0.05 level (2-tailed).

**Table 5:** Examining discriminant validity by dimension of SMILE-C and UES.

Dimension of SMILE-C	*r* (UES)
Diet and nutrition	0.20 *
Substance use	−0.06
Physical activities	0.26 **
Stress management	0.27 **
Restorative sleep	0.20 *
Social support	0.28 **
Environmental exposures (screen time/outdoor time)	0.02

** Correlation is significant at the 0.01 level (2-tailed); * correlation is significant at the 0.05 level (2-tailed).
